# Investigation on Natural Infection of Covert Mortality Nodavirus in Farmed Giant Freshwater Prawn (*Macrobrachium rosenbergii*)

**DOI:** 10.3390/ani12111370

**Published:** 2022-05-27

**Authors:** Jitao Xia, Chong Wang, Liang Yao, Wei Wang, Wenxiu Zhao, Tianchang Jia, Xingtong Yu, Guoliang Yang, Qingli Zhang

**Affiliations:** 1 College of Fisheries and Life Science, Shanghai Ocean University, Shanghai 201306, China; xiajitaook@126.com (J.X.); yaoliang2019@163.com (L.Y.); 13173113773@163.com (W.W.); wenxiuzhao2021@163.com (W.Z.); jiatc2021@163.com (T.J.); 2 Yellow Sea Fisheries Research Institute, Chinese Academy of Fishery Sciences, Function Laboratory for Marine Fisheries Science and Food Production Processes, Qingdao National Laboratory for Marine Science and Technology, Key Laboratory of Maricultural Organism Disease Control, Ministry of Agriculture, Qingdao Key Laboratory of Mariculture Epidemiology and Biosecurity, Qingdao 266071, China; wangchongyilin@163.com (C.W.); yuxingtong998@163.com (X.Y.); 3 College of Life Sciences, Huzhou University, Huzhou 313000, China; 4 Jiangsu Shufeng Prawn Breeding Co., LTD., Gaoyou 225600, China

**Keywords:** covert mortality nodavirus (CMNV), *Macrobrachium rosenbergii*, natural infection, histopathology

## Abstract

**Simple Summary:**

Covert mortality nodavirus (CMNV) is a newly discovered aquatic animal virus in recent years. Here, we detected CMNV positive in farmed giant freshwater prawn (*Macrobrachium rosenbergii*) from Jiangsu, China by TaqMan RT-qPCR. Meanwhile, in situ hybridization and histological analysis indicated that the intestine, gill, hepatopancreas and ovary of giant freshwater prawn were the target organs of CMNV. In addition, a large number of CMNV-like particles were observed in the hepatopancreas and gill tissues under transmission electron microscopy. Overall, our study confirms that giant freshwater prawn is a susceptible host of CMNV, further expands the known host range of CMNV, and provided a new direction for further investigation and exploration of multiple pathogenic factors of giant freshwater prawn disease.

**Abstract:**

Covert mortality nodavirus (CMNV), from the *Nodaviridae* family, is characterized by its unique cross-species transmission and wide epidemic distribution features. In this study, *Macrobrachium rosenbergii* was proved to be infected naturally by CMNV, which further expand the known host range of CMNV. Here, 61.9% (70/113) of the *M. rosenbergii* samples collected from Jiangsu Province were CMNV positive in the TaqMan RT-qPCR assay, which indicated the high prevalence of CMNV in *M. rosenbergii*. Meanwhile, the sequences of CMNV RdRp gene cloned from *M. rosenbergii* were highly identical to that of the original CMNV isolate from *Penaeus vannamei*. In situ hybridization (ISH) and histology analysis indicated that the intestine, gill, hepatopancreas and ovary were the targeted organs of CMNV infection in *M. rosenbergii*, and obvious histopathological damage including vacuolation and karyopyknosis were occurred in the above organs. Notably, the presence of CMNV in gonad alerted its potential risk of vertical transmission in *M. rosenbergii*. Additionally, numerous CMNV-like particles could be observed in tissues of hepatopancreas and gill under transmission electron microscopy. Collectively, our results call for concern of the potential negative impact of the spread and prevalence of CMNV in *M. rosenbergii* on its aquaculture, as well as providing a renewed orientation for further investigation and exploration of the diverse pathogenic factors causing *M. rosenbergii* diseases.

## 1. Introduction

Aquatic products are widely demanded and their production represents a part of economic growth, but their economic value will be reduced by aquaculture diseases. Diseases of aquatic products, such as viral infections of aquatic animals, have become more problematic and caused significant economic losses to the aquaculture industry [1]. Covert mortality nodavirus (CMNV), a shrimp pathogenic agent from the *Nodaviridae* family, has proved to possess a unique cross-species transmission feature [2,3]. While the vast majority of known viruses possess strong host specificity [4,5], owing to a variety of epidemiological, ecological and genetic variation factors, several emerging viruses occasionally conquer the bottleneck of the interspecies barriers and infect new hosts [6,7,8]. Furthermore, cross-species transmission of RNA viruses more easily occurred as their own internal instability and easy variation [9,10]. As a single-stranded RNA virus isolated from shrimp [2], CMNV had been proved that its host range performed extremely broad [11]. In addition to shrimp, it could also naturally infect other crustaceans (such as a hermit crab *Diogenes edwardsii*, a ghost crab *Lepidopa benedicti* and a fiddler crab *Tubuca arcuata*) [12] and several teleostean fishes, including goldfish *Carassius auratus* [13], Japanese flounder *Paralichthys olivaceus* [14], gobiid fish *Mugilogobius abei* [15], zebrafish *Danio rerio* [16], and small yellow croaker *Larimichthys polyactis* [17]. Moreover, sea cucumber (*Apostichopus japonicus*), a species of Echinodermata, has been confirmed as one of the susceptive hosts of CMNV [3,11]. Despite these advances in CMNV’s host range, facing such extraordinary capacity of cross-host transmission of CMNV, its host spectrum still needs our further investigation to extend our understanding of the potential risks of aquaculture caused by CMNV.

*Macrobrachium rosenbergii*, also named the giant freshwater prawn, is one of the vital species in many freshwater ecosystems, and also a valuable crustacean species possessing high economic value in Asian aquaculture [18,19,20]. Some viruses emerged that cause serious economic losses in the intensified aquacultured *M. rosenbergii* [21]. For instance, *Macrobrachium rosenbergii* nodavirus (MrNV) along with extra small virus (XSV), decapod iridescent virus 1 (DIV1), and white spot syndrome virus (WSSV) could all infect *M. rosenbergii* and cause serious diseases [18,22]. *M. rosenbergii* was once suspected to be one of susceptive hosts of CMNV when it was detected as CMNV positive in the RT-LAMP assay [23]. Up to now, evidence of natural infection of CMNV in *M. rosenbergii*, or whether it can be infected by CMNV or not is still needed for further investigation.

Here, the present study was designed to accurately investigate the infection and prevalence of CMNV in *M. rosenbergii* and analyzed the histopathological changes caused by CMNV using TaqMan RT-qPCR, histopathology, in situ hybridization (ISH) and ultrastructural observation assays. The results will be helpful for the farmers to strength the prevention and control of CMNV infection in *M. rosenbergii* and to avoid the potential huge economic losses caused by the widespread prevalence of CMNV. 

## 2. Materials and Methods

### 2.1. Sample Collection

A total of 113 live *M. rosenbergii* samples (body length 12–14 cm) were collected from local farmed ponds in Gaoyou, Jiangsu Province at the time periods of 26 June, 17–25 September, and 18–19 October 2021. Additionally, the samples collected on 26 June 2021 exhibited disease signs such as abnormal swimming, empty intestine and shell softening. In the progress of sampling, the hepatopancreas, intestine, gill and gonad tissues of these prawns were sampled and cut into three parts: one part was fixed in 2.5% glutaraldehyde solution (Solarbio, Beijing, China) for transmission electron microscopic (TEM) examination; the second part was fixed in 4% paraformaldehyde solution (Sinopharm, Beijing, China) for ISH detection and histopathological analysis; the third part was chopped up and kept in RNAstore solution (Tiangen, Beijing, China) and 95% ethanol (Sinopharm, Beijing, China) for molecular pathogen identification.

### 2.2. Total RNA and DNA Purification

Total RNA was prepared from RNAstore-preserved *M. rosenbergii* tissues (approximately 50 mg) using the commercial RNA extraction kit (Takara, Dalian, China) following the manufacturer’s instructions. The detailed protocols of the extraction of the tissue RNA were performed as previously described [11]. 

Total DNA was extracted from *M. rosenbergii* tissues (approximately 50 mg) preserved in 95% ethanol using the TIANamp Marine Animal DNA Kit (Tiangen Biotechnology, Beijing, China) according to the manufacturer’s instructions.

### 2.3. Detection of Pathogens in M. rosenbergii Samples

The extracted total RNAs or DNAs of *M. rosenbergii* were used as PCR detection templates to detect the nine common pathogens, including white spot syndrome virus (WSSV), infectious hypodermal and hematopoietic necrosis virus (IHHNV), Enterocytozoon hepatopenaei (EHP), acute hepatopancreatic necrosis disease causing *Vibrio* (*Vp*_AHPND_), decapod iridescent virus 1 (DIV1), Taura syndrome virus (TSV), yellow head virus 1 (YHV-1), infectious myonecrosis virus (IMNV), and covert mortality nodavirus (CMNV), according to the OIE Manual and previous reported methods [18,24]. Eyestalks, gills, heart, gonads, intestines, muscles and appendages were assayed for CMNV load in tissues of *M.* *rosenbergii* by TaqMan RT-qPCR. The above PCR primer sequences, probe sequences and procedures for CMNV are listed in Tables S1 and S2.

### 2.4. Sequencing of CMNV Amplicons and Their Phylogenetic Analysis

RNA1 of CMNV was amplified and cloned by using the PCR primers in Table S3. The amplified products were sequenced at commercial sequencing company of Sangon Biological Engineering (Shanghai, China) Co. Ltd. Subsequently, sequences were aligned with those of 26 relevant RdRp sequences of Nodavirus species obtained from the GenBank database (Table 1) by BLAST. Finally, the phylogenetic tree was constructed by using the MEGA 6.0 [25] via default settings, and then edited by using the online tool Interactive Tree of Life (iTOL) (https://itol.embl.de/, accessed on 23 October 2021).

### 2.5. Analysis of ISH and Histopathology of CMNV-Positive M. rosenbergii Individuals

Tissue samples of intestine, hepatopancreas, gill and gonad from partial CMNV-positive *M. rosenbergii* individuals which had been fixed in 4% PFA for 24 h, are dehydrated in a graded series of 70%, 85%, 90%, 95% and absolute ethanol, then processed and embedded in paraffin [26]. Two pieces of paraffin slices (3 µm) from each tissue samples were prepared using a rotary microtome (Leica, Wetzlar, Germany): one section was used for ISH detection [3,14,23]; another was stained with hematoxylin and eosin (H&E) as descripted elsewhere [27] for histopathological analysis. All slides of ISH and H&E were digitized with the whole-slide Pannoramic MIDI scanner (3DHISTECH Ltd., Budapest, Hungary) at 40× magnification.

### 2.6. Observation of CMNV by Transmission Electron Microscopy (TEM)

We performed the TEM observation of CMNV positive individuals detected by ISH. The tissue blocks (volume approximately 1 mm^3^) preserved in 2.5% glutaraldehyde were fixed for 2 h with 1% osmium tetroxide, then embedded in epoxy resin [28,29]. Next, the ultra-thin sections (50 nm) were prepared according to previous reports by an ultramicrotome (Leica EM UC7) and stained with uranyl acetate [30,31]. Finally, the samples were carried on copper grids and examined using TEM (HT7700, Hitachi, Tokyo, Japan) at 100 kV.

## 3. Results

### 3.1. Molecular Detection of CMNV in M. rosenbergii

All samples of 27 *M. rosenbergii* samples, showing typical symptoms such as abnormal swimming, empty intestine and shell softening, sampling on 26 June 2021, were tested to be negative for WSSV, IHHNV, EHP, *Vp*_AHPND_, DIV1, TSV, YHV, and IMNV, but to be positive for CMNV (Table S4). According the TaqMan RT-qPCR assay, 70 out of 113 *M. rosenbergii* individuals were CMNV positive, and 44 out of 50 individuals were CMNV positive through ISH detection (Table 2). Meanwhile, CMNV load was detected in 42 RNAs from 6 CMNV-positive *M. rosenbergii* (Table 3). The CMNV load in gonad of *M. rosenbergii* samples with typical clinical syndromes was the highest compared to the other seven tissues tested averaged 10^4.41 ± 0.23^ Copies/µg. CMNV loads were also high in intestines and muscles samples, both exceeding 10^3.7^ copies/µg. The CMNV loads in appendages, gills, heart and eyestalks were almost identical averaging 10^2.86 ± 0.28^, 10^2.68 ± 0.76^, 10^2.43 ± 0.81^ and 10^2.27 ± 0.50^, respectively.

### 3.2. Phylogenetic Analysis of CMNV in M. rosenbergii

**Figure 1:**
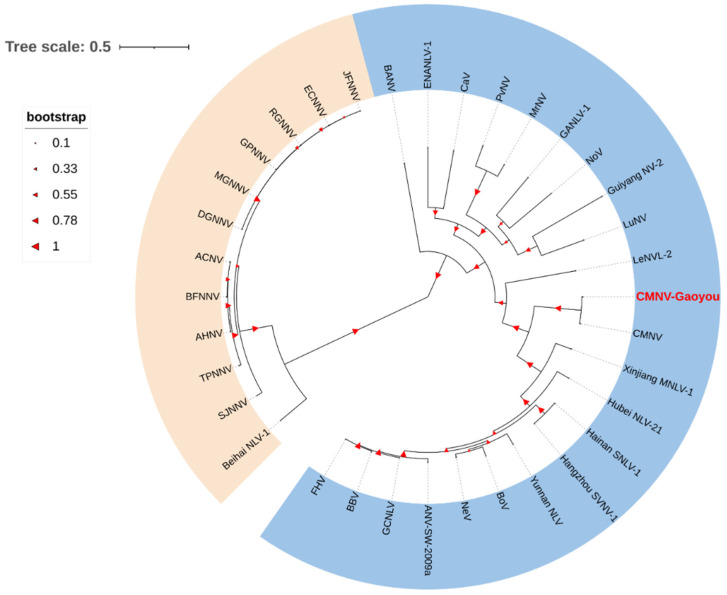
Phylogenetic tree analysis of the RdRp amino acid sequences of both covert mortality nodavirus (CMNV) isolated from *Macrobrachium rosenbergii* samples and other Nodavirus species (Table 1). CMNV isolate of *M. rosenbergii* sampled from Gaoyou, Jiangsu province is marked by red font. Species of *Alphanodavirus* and *Betanodavirus* are shown in blue and orange background, respectively. The phylogenetic tree was built using the MEGA 6.0 program based on the neighbor-joining method. The scale bar is 0.5. The bootstrap value was 1000 replicates, and the larger the bootstrap value, the higher the confidence of the branch.

The sequence (3228 bp, GenBank no. ON209169) of RNA1 amplification product of CMNV derived from *M. rosenbergii* shared 98.8% identity with the RdRp gene (GenBank no. QQP17176.1) of the initial CMNV isolate. Analysis of phylogenetic tree showed that the RdRp-deduced amino acids sequence of CMNV-Gaoyou isolated from *M. rosenbergii* were assigned into the branch that has the CMNV original-sequence, which belonged to the *Alphanodavirus* that was phylogenetically different from the *Betanodavirus* members (Figure 1). 

### 3.3. Detection of Sites of CMNV Natural Infection in M. rosenbergii Tissues Using ISH Assay

**Figure 2:**
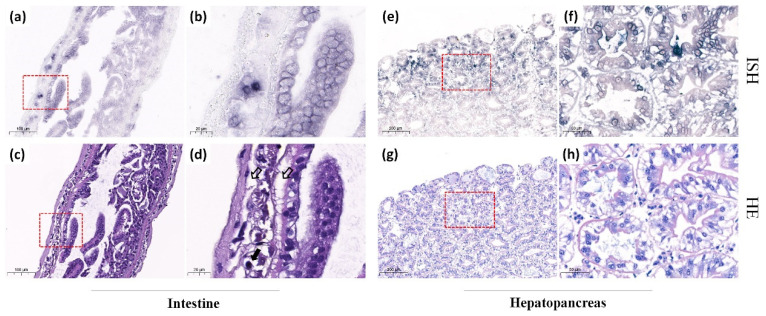
Micrographs of histopathological and in situ hybridization (ISH) assays for the intestine and hepatopancreas from *Macrobrachium rosenbergii* naturally infected by covert mortality nodavirus (CMNV). (**a**) ISH micrographs of intestine. (**b**) Magnified views of the red-framed areas of (**a**). Note the bluish-purple hybridization signals of CMNV probe. (**c**) H&E staining micrographs of intestine histological section. (**d**) Magnified views of the red-framed areas of (**c**). Karyopyknosis (thick black arrows) and extensive vacuolation (hollow arrows) were observed in the intestinal muscle layer. (**e**) ISH micrographs of hepatopancreas. (**f**) Magnified views of the red-framed areas of (**e**). Note the bluish-purple CMNV positive hybridization signals. (**g**) H&E staining micrographs of hepatopancreas histology section. (**h**) Magnified views of the red-framed areas of (**g**). Note the necrosis and slightly exfoliated hepatopancreatic tubular epithelium cells. Scale bars: (**a**,**c**) 100 µm, (**b**,**d**) 20 µm. (**e**,**g**) 200 µm, (**f**,**h**) 50 µm.

**Figure 3:**
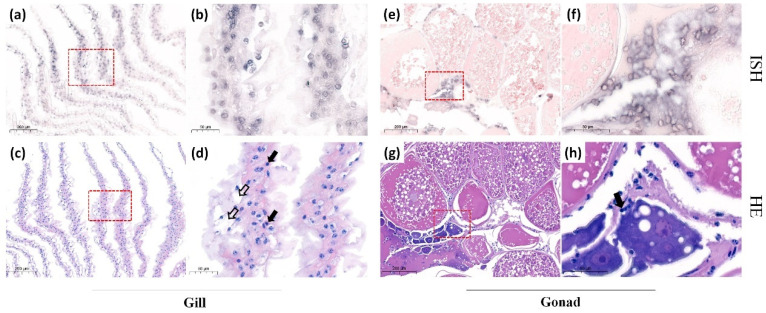
Micrographs of histopathological and in situ hybridization (ISH) assays for the gill and gonad from *Macrobrachium rosenbergii* naturally infected by covert mortality nodavirus (CMNV). (**a**) ISH micrographs of gill. (**b**) Magnified view of the red-framed areas of (**a**). Note the bluish-purple hybridization signals of CMNV probe. (**c**) H&E staining micrographs of gill histological section. (**d**) Magnified view of the red-framed areas of (**c**). Note the gill tissue edema, extensive vacuolation (hollow arrows) and karyopyknosis (thick black arrows). (**e**) ISH micrographs of gonad histological section. (**f**) Magnified view of the red-framed areas of (**e**). Note the bluish-purple CMNV positive hybridization signals. (**g**) H&E staining micrographs of gonad histological section. (**h**) Magnified view of the red-framed areas of (**g**). Karyopyknosis (thick black arrows) and hemocytes infiltration could be observed in ovary. Scale bars: (**a**,**c**,**e**,**g**) 200 µm, (**b**,**d**,**f**,**h**) 50 µm.

The ISH analysis was performed on *M. rosenbergii* that were determined to be CMNV-positive using TaqMan RT-qPCR. The ISH results revealed that widespread bluish-purple CMNV-positive hybridization signals in the intestine (Figure 2a,b) and hepatopancreas (Figure 2e,f). Additionally, the CMNV-positive signals could also be present in gill (Figure 3a,b) and ovary (Figure 3e,f) tissues.

### 3.4. Histopathological Analysis of CMNV-Positive Individuals in ISH Assay

The results of histological examination revealed that serious pathological changes occurred in tissues of CMNV ISH positive individuals. Extensive vacuolation as well as karyopyknosis could be observed in epithelial cells of intestine (Figure 2c,d) where the CMNV ISH positive signals presented. Moreover, the exfoliated hepatopancreatic tubular epithelium cells could also be noted in hepatopancreas tissue (Figure 2g,h). The gill exhibited abnormal histopathological changes, such as the severely edematous gill filament, vacuolation and karyopyknosis (Figure 3c,d). Notably, ovary sections showed some pathological changes including hemocytes infiltration and karyopyknosis (Figure 3g,h).

### 3.5. Detection of CMNV Infection in M. rosenbergii by TEM Assay

**Figure 4:**
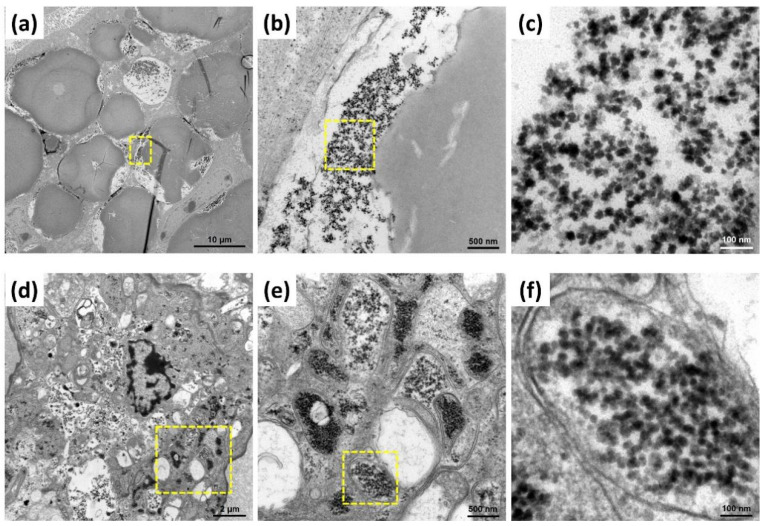
Transmission electron microscope (TEM) micrographs of hepatopancreas and gill ultrathin section of *Macrobrachium rosenbergii* naturally infected with covert mortality nodavirus (CMNV). (**a**–**c**) show the TEM micrographs of hepatopancreas ultrathin section; (**d**–**f**) show the TEM micrographs of gill ultrathin section. (**b**,**c**) and (**e**,**f**) show magnified micrographs in the yellow-framed areas of (**a**,**b**) and (**d**,**e**), respectively. Note the high number of CMNV-like virus particles in (**c**,**f**).

In order to further confirmation CMNV infection status in *M. rosenbergii* samples determined to be CMNV positive by TaqMan RT-qPCR and ISH, ultrathin sections of these samples were examined. The result showed that obvious inclusion bodies were present both in hepatopancreas and gill tissues of *M. rosenbergii* sample and abundant CMNV-like particles (about 28–32 nm in diameter) could be observed clearly in the inclusions of hepatopancreas and gill (Figure 4).

## 4. Discussion

In the past decades, Aquaculture is considered among the fastest-growing sector producing animal protein in the world, and contributes significantly to global food security [32,33]. The market demand for marine and freshwater aquacultured shrimp is also increasing [18]. *M. rosenbergii* has been regarded as one of the major species of farming freshwater prawns both in China and the Southeast Asia countries. This increasing market demand for *M. rosenbergii* supported the continuous expansion and development of high breeding densities farming of *M. rosenbergii*, leading to the increased occurrence of diseases of *M. rosenbergii* and significant economic losses to a large extent [20]. Two viral diseases had been reported in *M. rosenbergii* [20] including white tail disease (WTD) and “white head” disease, which are caused by *M. rosenbergii* nodavirus (MrNV) and decapod iridescent virus 1 (DIV1) [18], respectively. Lethargy, obvious muscle whiteness of post-larvae prawn and pale hematopoietic organ are typical clinical symptoms of the WTD and white head diseases, respectively [18,34,35]. However, in this research, the diseased *M. rosenbergii* samples obtained from Jiangsu province showed disease signs such as abnormal swimming, empty intestine and shell softening, which were very similar to the clinical signs of CMNV infection in *Penaeus vannamei* [2,3]. So far, there are no reports of CMNV infection in *M. rosenbergii*. Meanwhile, combined with the previous RT-LAMP positive results of CMNV in *M. rosenbergii* [23], it drove us to further investigate the prevalence of this virus in the farmed *M. rosenbergii*, as well as to reveal histopathological changes caused by CMNV.

The TaqMan RT-qPCR detection results showed that among all the 113 *M. rosenbergii* samples collected from Gaoyou, Jiangsu Province, 61.9% (70/113) of individuals were found to be CMNV positive. Meanwhile, the sequences of the sub-genomic RNA1 fragment (RdRp gene) of the CMNV isolates from the *M. rosenbergii* samples were highly identical to the CMNV original RdRp gene, which supplied further evidence that *M. rosenbergii* of this study indeed infected with CMNV. Additionally, 50 CMNV positive samples determined by TaqMan RT-qPCR were randomly selected for CMNV ISH assay, and 88.0% (44/50) samples were also tested to be CMNV positive in the ISH assay. The reason for the inconsistency between ISH and TaqMan RT-qPCR results might be caused by the different detection sensitivity of these two assays. As we know, the CMNV detection sensitivity of the TaqMan RT-qPCR, which can detect viral single copy, possesses much higher detection sensitivity than ISH. This is the first report that proved CMNV naturally infects the aquaculture *M. rosenbergii*, and the high infection rate of CMNV in these samples revealed the potential risk of this virus causing outbreaks of disease in farmed *M. rosenbergii*.

To further explore the targeted organs of CMNV infection in *M. rosenbergii*, more tissues of CMNV-positive *M. rosenbergii* were selected to be analyzed by ISH and H&E staining in this study. CMNV-positive signals were detected in intestine, gill, hepatopancreas and ovary tissues. Simultaneously, obvious histopathological lesions were presented in the same sites where hybridization signals occurred, including karyopyknosis and vacuolation in intestinal muscle layer, gill and ovary, as well as the exfoliated hepatopancreatic tubular epithelium cells. Furthermore, a large number of CMNV-like virus particles were observed in the hepatopancreas and gill tissues under the TEM. All these results provided evidence of the broad range tissue tropism of CMNV infection in *M. rosenbergii*, and the results revealed that CMNV infection can lead to severe pathological damage to most of the target tissues. Weakening resistance of *M. rosenbergii* and insufficient biosecurity measures implemented in *M. rosenbergii* farming may reduce the capacity of adaptation to environment and increase the pathogens exposing risks, which might be the reason of the rising viral diseases in *M. rosenbergii* aquaculture industry in recent years. Certainly, mortality and perniciousness of *M. rosenbergii* disease caused by CMNV is worthy of further investigation.

This study also revealed CMNV infection in ovary tissues, indicating that CMNV may be transmitted vertically in *M. rosenbergii*. It was previously reported that CMNV could be vertically transmitted from the male or female parent to the offspring in *Exopalaemon carinicauda* [36]. Moreover, CMNV infection also presented in gonad of zebrafish, sea cucumber and small yellow croaker [3,16,17]. Therefore, the natural CMNV infection in the ovary of *M. rosenbergii* may increase the risk of CMNV spread through larva in farmed *M. rosenbergii*. CMNV infection of gonadal tissue in *E. carinicauda* will lead to difficulty in hatching fertilized eggs and reduced survival rate of larvae [36]. Therefore, in the future, more attention should be paid to the impact of CMNV infection on the reproductive performance and seed production of *M. rosenbergii*, so as to provide the theoretical basis for eliminating CMNV hazards in the aquaculture industry of *M. rosenbergii*.

## 5. Conclusions

This study showed that *M. rosenbergii* is a new sensitive host of CMNV, and CMNV displayed an extremely broad range tissue tropism in this new host. The result of pathology suggested that CMNV might be a potential pathogen causing diseases in farmed *M. rosenbergii.* Since CMNV-like virus particles were observed in the gonads, it is suspected that CMNV might be transmitted vertically in *M. rosenbergii*. Meanwhile, considering the high natural infection rate of CMNV in *M. rosenbergii* demonstrated in this research, more attention should be paid to the potential negative impact of CMNV spread and prevalence in *M. rosenbergii* in aquaculture systems. Taken together, this study supports a renewed orientation for further investigation and exploration of the diversity of pathogenic agents in farming *M. rosenbergii*.

## Figures and Tables

**Table 1:** Names and abbreviations of *Nodaviridae* species used in phylogenetic tree.

Virus	Abbreviation	GenBank No.
Atlantic cod nodavirus	ACNV	ABR23188.1
Atlantic halibut nodavirus	AHNV	AAY34458.1
Barfin flounder nervous necrosis virus	BFNNV	YP_003288756.1
Bat associated nodavirus	BANV	QOR29565.1
Beihai noda-like virus 1 strain	Beihai NLV-1	YP_009333380.1
Black beetle virus	BBV	YP_053043.1
Boolarra virus	BoV	NP_689439.1
Carano virus	CaV	BCG55383.1
Covert mortality nodavirus	CMNV	QQP17175.1
Dragon grouper nervous necrosis virus	DGNNV	AAU85148.1
*Drosophila melanogaster* American nodavirus (ANV) strain SW-2009a	DmANV-SW-2009a	ACU32794.1
Epinephelus coioides nervous necrosis virus	ECNNV	AXP99039.1
Flock House virus	FHV	NP_689444.1
Golden pompano nervous necrosis virus	GPNNV	AEK48150.1
Grapevine-associated noda-like virus 1	GANLV-1	QXN75416.1
Guiyang nodavirus 2	Guiyang NV-2	UHK03035.1
Gungahlin Chrysomya noda-like virus	GCNLV	QIJ70031.1
Hainan sediment noda-like virus 1	Hainan SNLV-1	QYF49900.1
Hangzhou sepedon violaceus nodavirus 1	Hangzhou SVNV-1	UHR49743.1
Hubei noda-like virus 21 strain	Hubei NLV-21	APG76486.1
Japanese flounder nervous necrosis virus	JFNNV	ACN58225.1
Lutzomyia nodavirus	LuNV	AKP18615.1
*Macrobrachium rosenbergii* nodavirus	MrNV	AAQ83832.1
Mouse grouper Nervous Necrosis Virus	MGNNV	AEK48140.1
Newington virus	NeV	AMO03244.1
Nodamura virus	NoV	NP_077730.1
*Penaeus vannamei* nodavirus	PvNV	YP_004207810.1
Redspotted grouper nervous necrosis virus	RGNNV	YP_611155.1
Striped jack nervous necrosis virus	SJNNV	BAB64329.1
Tiger puffer nervous necrosis virus	TPNNV	YP_003288759.1
Xinjiang mountain noda-like virus 1	Xinjiang MNLV-1	QYF49916.1
Yunnan noda-like virus	Yunnan NLV	QYF49925.1

GenBank No. indicate the GenBank accession numbers of the sequences of deduced amino acid of RNA-dependent RNA polymerase from different Nodaviruses.

**Table 2:** Detection of CMNV of *M. rosenbergii* samples collected from shrimp ponds in Gaoyou.

CMNV Detection Methods	Detection Rate of CMNV Positive
TaqMan RT-qPCR ISH	65.9% (70/113) 88.0% (44/50)

**Table 3:** CMNV copies in different tissues detected in CMNV-positive *M. rosenbergii*.

Tissue	Number	Mean (Copies/µg-RNA)
Gonads	6	10^4.41^^±^^0.23^
Intestines	6	10^3.79^^±^^0.30^
Muscles	6	10^3.71^^±^^0.17^
Appendages	6	10^2.86^^±^^0.28^
Gills	6	10^2.68^^±^^0.76^
Heart	6	10^2.43^^±^^0.81^
Eyestalks	6	10^2.27^ ^± 0.50^

## Data Availability

Data available on request from the authors.

## Supplementary Materials

The following supporting information can be downloaded at: https://www.mdpi.com/article/10.3390/ani12111370/s1, Table S1: Primers and the TaqMan probe for CMNV RT-qPCR; Table S2: The program for TaqMan RT-qPCR of CMNV; Table S3: PCR primer’s sequence used to amplify RNA1 of CMNV; Table S4: Common pathogens test results of *Macrobrachium rosenbergii* samples collected at 26 June 2021; Figure S1: The negative control picture for ISH assay.
