# Supplementary material for: Investigation on Natural Infection of Covert Mortality Nodavirus in Farmed Giant Freshwater Prawn (Macrobrachium rosenbergii)
Source: Animals (Basel). 2022 May 27;12(11):1370. doi: 10.3390/ani12111370 (PMC9179840; doi:10.3390/ani12111370)
Supplement: Supplementary file 1 [file animals-12-01370-s001.zip › animals-1508281-supplementary.pdf]

Table S1. Primers and the TaqMan probe for CMNV RT-qPCR

Name	Sequences (5′-3′)	Length of amplicons
CMNV-Taq-F	CGAGCTAATCCAAGCACTTC	158 bp
CMNV-Taq-R	ACCTGTTAGGTACGCTACCA	
TaqMan probe	FAM CGCTCACGGCTTTGGATACCTT TAMRA	

Table S2. The program for TaqMan RT-qPCR of CMNV

Temperature	Time	Cycle
51℃	15 min	1
95℃	5 min	1
94℃	10 s	40
52.7℃	30 s	40

Table S3. PCR primer's sequence used to amplify RNA1 of CMNV

Primer name	Primer sequences (5′-3′)	Length of amplicons
RNA1-5W-F1	TGCTGACAGCGAGGTT	1228 bp
RNA1-5W-R1	TGGGCTTCAGGGTTTT	
RNA1-5W-F4	TCTGTAACATCTGACGTG	367 bp
RNA1-5W-R4	CCTCATCAATCATTCG	
RNA1-3S-F1	GCCTTGGCTTAACTGC	2399 bp
RNA1-3S-R1	GGTCCTTTACTGGGTCTC	

Figure S1. The negative control picture for ISH assay

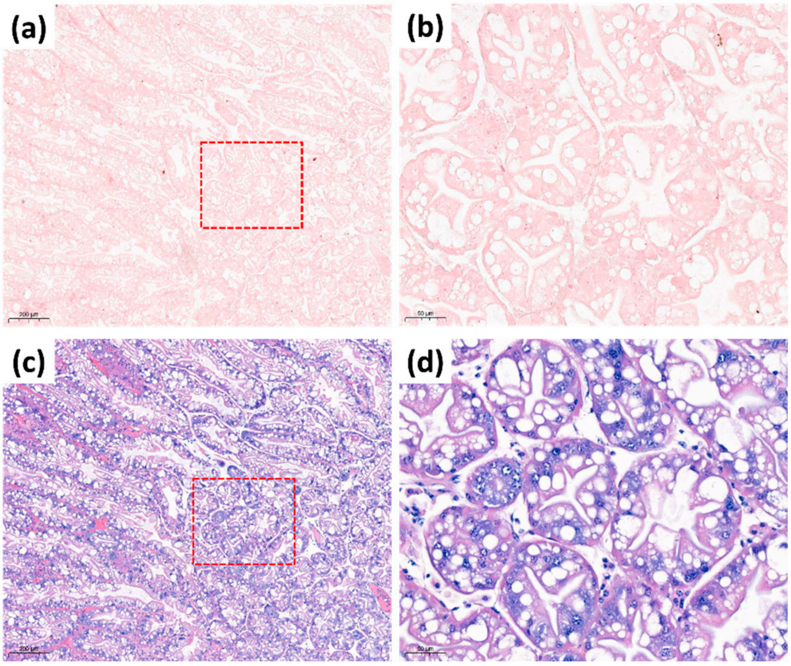

Figure S1. Micrographs of hepatopancreas from *Macrobrachium rosenbergii* that uninfected with covert mortality nodavirus (CMNV) in the histopathological and in situ hybridization (ISH) assays. (a) ISH micrographs of hepatopancreas. (b) Magnified views of the red framed areas of (a). Bluish-purple

hybridization signals were not detected. (c) H&E staining micrographs of hepatopancreas. (d) Magnified views of the red-framed areas of (c). Scale bars: (a, c) 200 μm, (b, d) 50 μm.

Table S4. Common pathogens test results of *Macrobrachium rosenbergii* samples collected at June 26, 2021.

Samples	WSSV	IHHNV-309	IHHNV-389	EHP	V_{AHPND}	DIV1	TSV	YHV-1	IMNV	CMNV
	N-PCR	PCR	PCR	N-PCR	N-PCR	N-PCR	RT-PCR	RT-N-PCR	RT-N-PCR	ISH
20210626040		-	-	-	-	-	-	-	-	+
20210626041	-	-	-	-	-	-	-	-	-	+
20210626042	-	-	-	-	-	-	-	-	-	+
20210626043	-	-	-	-	-	-	-	-	-	++
20210626044	-	-	-	-	-	-	-	-	-	++
20210626045	-	-	-	-	-	-	-	-	-	+
20210626046	-	-	-	-	-	-	-	-	-	+
20210626047	-	-	-	-	-	-	-	-	-	+
20210626048	-	-	-	-	-	-	-	-	-	++
20210626049	-	-	-	-	-	-	-	-	-	+
20210626050	-	-	-	-	-	-	-	-	-	+
20210626051	-	-	-	-	-	-	-	-	-	++
20210626052	-	-	-	-	-	-	-	-	-	+
20210626053	-	-	-	-	-	-	-	-	-	++
20210626054	-	-	-	-	-	-	-	-	-	+++
20210626055	-	-	-	-	-	-	-	-	-	+
20210626056	-	-	-	-	-	-	-	-	-	+

Note: “-“ indicates negative; “++” indicates positive; “+++” indicates strong positive.
